# Selective reduction of visceral adipose tissue with injectable ice slurry

**DOI:** 10.1038/s41598-023-43220-9

**Published:** 2023-09-28

**Authors:** Sara Moradi Tuchayi, Yeva Khachatryan, Ying Wang, R. Rox Anderson, Jialiang S. Wang, Marc N. Wein, Lilit Garibyan

**Affiliations:** 1 Wellman Center for Photomedicine, Massachusetts General Hospital, 50 Blossom Street-Thier 2, Boston, MA 02114 USA; 2 Department of Dermatology, Harvard Medical School, Boston, USA; 3 Endocrine Unit, Massachusetts General Hospital, Harvard Medical School, Boston, USA

**Keywords:** Metabolic syndrome, Obesity

## Abstract

Reduction in visceral adipose tissue (VAT) mass reduces body weight and metabolic disease risk in obese patients. However surgical removal of VAT is highly invasive and thus not clinically feasible. We developed an injectable ice slurry for selective reduction of adipose tissue through cryolipolysis. The aim of this study was to investigate safety, feasibility and mechanism of ice slurry-induced cryolipolysis of VAT. Perigonadal VAT in diet-induced obese mice and rats was subjected to slurry or sham treatment. Body weight and blood chemistry were monitored for 56 days post-treatment. Histological analysis and molecular studies were performed to elucidate mechanisms of fat reduction. Treatment of VAT was well tolerated in all animals. Slurry induced adipocyte cell death via selective cryolipolysis; significant weight loss was noted at day 21 post-treatment. RNA sequencing from treated VAT samples showed increased expression of genes involved in inflammation, immune response, collagen biosynthesis and wound healing, and decreased expression of adipokines. This study demonstrates that slurry treatment is safe and effective in inducing cryolipolysis of VAT and subsequent weight loss in mice. Ice slurry is promising as a minimally-invasive treatment to reduce visceral adipose tissue.

## Introduction

Obesity is a global epidemic. According to the World Heath Organization at least 2.8 million people die each year from being overweight or obese^1^. Obesity and overweight are defined by having excessive or abnormal fat accumulation that may impair health. Obesity is associated with numerous comorbidities that form metabolic syndrome and cause significant morbidity and mortality^2^. While obesity is characterized by enlargement of both visceral and subcutaneous adipose tissues, the visceral adipose tissue (VAT) is more strongly associated with development of the metabolic syndrome than subcutaneous adipose tissue^2,3^. Abdominal visceral obesity, the marker of VAT excess, is the major risk factor for systemic inflammation, insulin resistance, metabolic syndrome and cardiovascular disease^4,5^.

Weight loss results in reduced volume of both visceral and subcutaneous adipose tissues and reduces obesity comorbidities^6^. Current weight loss treatments include diet and lifestyle modification, anti-obesity medications, bariatric surgery, and implantable devices^7^. Adherence to diet and lifestyle treatments is challenging for most patients^8^. Although recent advances in anti-obesity medications are promising, these medications are costly and they are effective only for the duration of the therapy. In addition, these medications are associated with side effects and in many cases even contraindicated^9^. Invasie surgical procedures are associated with significant risk^10^. Most importantly, maintenance of weight loss regardless of the method used is difficult for most patients in long term^8^.

It is known that weight loss by reduction in abdominal VAT mass improves metabolic profile^4^. Intra-abdominal lipectomy has been shown to reverse insulin resistance in obese rodents and obese patients^11–14^. However, surgical removal of VAT is highly invasive thus not clinically practical. A simple method of inducing selective VAT loss without surgery or systemic side effects could be a desirable treatment in obese patients.

Adipose tissue is selectively susceptible to cold injury^15–17^. Our laboratory was the first to demonstrate that controlled topical cooling of adipose tissue induces subcutaneous fat reduction via cryolipolysis^18,19^. Selective cryolipolysis with topical cooling has been widely used in patients to reduce subcutaneous fat in various body parts^20,21^, and studies have not shown any changes in serum lipids or markers of liver function^22,23^. Although this non-invasive method of fat reduction is appropriate for targeting subcutaneous fat, VAT is well beyond the depth limit for treatment by topical cooling. We recently developed a novel method of selective fat reduction using a biocompatible injectable ice slurry^24^. Similar to topical cooling, slurry is able to induce cryolipolysis and selective reduction in subcutaneous adipose tissue without any damage to surrounding tissues^24^. Injection of slurry in subcutaneous adipose tissue was also shown to be safe in human subjects and effective in inducing cryolipolysis^25^. This simple and injectable method of selective fat reduction can potentially allow adipose tissue at any anatomic location to be targeted, thus expanding the medical indications and applications for which selective cryolipolysis can be used. In this study, we hypothesized that injectable ice slurry can be used for safe and effective reduction of VAT and lead to weight loss in obese mice. Using histological and transcriptomic studies we also sought to determine the potential mechanism of slurry induced VAT reduction.

## Methods

### Slurry production

Slurry composed of normal saline and 10% glycerol was made with a sterilized commercial slurry maker (Vollrath Frozen Beverage Dispenser; Vollrath Co, LLC, Sheboygan, WI) and a prototype sterile device (Sage Product Development Inc, Foxborough MA).

### Animal experiments

Animal studies were approved by Massachusetts General Hospital Institutional Animal Care and Use Committee (IACUC). All experiments were performed in accordance with the Guide for the Care and Use of Laboratory Animals published by the US National Institutes of Health. Authors complied with the ARRIVE guidelines. For mouse experiments, adult male diet-induced obese C57BL/6J mice 38-53g and 19 weeks old were purchased from the Jackson Laboratory (Bar Harbor, ME) and housed at MGH in accordance with animal care regulations. Animals were fed with the same high fat diet (rodent diet with 60 kcal% fat) for the duration of study. Twenty-seven mice were used in this study. Perigonadal adipose tissue on one side was exposed through an abdominal incision and it was submerged in slurry or room temperature control solution (melted slurry solution at 20 °C) for 10 min. Slurry treatment in mice was performed by topical application due to limitation of volume that can be injected into the mice without causing volume overload. Abdominal incision was closed with sutures after treatment. Body weight of animals was monitored for duration of the study. Animals were sacrificed at day 1, day 21, and day 56 post treatment. In comparison to mice, rats tolerate larger volumes of injection without becoming volume overloaded. Therefore, the slurry injection studies were performed in a rat model of obesity. For rat experiments, adult male Sprague-Daley rats 200g and 8 weeks old were purchased from the Charles River Laboratories (Wilmington, MA) and housed at MGH in accordance with animal care regulations. Animals were fed with high fat diet (rodent diet with 60 kcal% fat) for 9 weeks prior to the treatment and then for the duration of study. Twenty rats were used in this study. Perigonadal adipose tissue on one side was exposed through an abdominal incision, and injected with 10 ml of slurry prior to closing the incision with sutures. The untreated side served as control. To test the safety of slurry injections on kidney and liver function, and on lipid profile, blood samples were taken from rats treated with slurry at baseline and at different time points post treatment as mentioned above.

Animals were sacrificed at 24 h, 3 days, 7 days, 14 days, 1 month, and 2 months after treatment and samples were taken for histology and molecular studies. Carbon dioxide overdose followed by bilateral thoracotomy as secondary physical method was used as the method of euthanization for all animals in this study.

### Histology

Samples of perigonadal adipose tissue were fixed in 10% formalin, then processed and embedded in paraffin, and sectioned at 5 μm, deparaffinized and stained with hematoxylin and eosin (H&E). TUNEL staining of paraffin sections was done using Promega™ DeadEnd™ Fluorometric TUNEL System kit according to the manufacturer’s instructions (Promega, Madison, WI). A board-certified pathologist blindly assessed the biopsy samples for histologic changes.

### RNA-seq

RNA-seq libraries were constructed from total RNA using polyA selection followed by NEBNext UltraDirectional kit workflow (New England Biolabs, MA) and sequenced on the Illumina HiSeq 2500 instrument, resulting in approximately 30 million reads per sample on average. N = 3 biologic replicates were performed for each condition. Raw sequencing data were performed with quality control using FastQC/0.11.3. Transcripts were quantified using Salmon^26^. Salmon index for rat was generated based on the Ensembl fasta file for mRatBN7.2 (release 108). Differential expression analysis was performed using DESeq2^27^ package based on the criteria of false discovery rates (FDR) < 0.05.

Volcano plots and heatmaps were made using SRPlot (http://www.bioinformatics.com.cn/srplot) and ggplot2/tidyverse packages from Bioconductor and tidyverse (https://github.com/kevinblighe/EnhancedVolcano, https://ggplot2.tidyverse.org). Gene Ontology enrichment analysis was performed with Metascape^28^.

### Quantitative RT-PCR

To determine the mechanism of VAT tissue reduction based on the histological findings and RNA-seq data, we used quantitative RT-PCR to examine the expression level of pro-inflammatory cytokines that are known to play a role in the inflammatory response in adipose tissue and subsequent normal wound healing, including IL-6, IL-1 b, GM-CSF, MCP-1, IL-1 a, IL-10 and TNF a. Quantitative RT-PCR was used to assess the expression level of mRNA in perigonadal adipose tissue samples in mice at 1 day post treatment. TissueLyser II (Qiagen, Germantown, MD) was used to homogenize samples in Qiazol Lysis Reagent (Qiagen, Germantown, MD). RNeasy Lipid Tissue Mini Kit (Qiagen, Germany) was used for RNA isolation. To make cDNA, High-Capacity RNA-to-cDNA Kit (Applied Biosystems, Foster city, CA) was used with 1 μg of total RNA. For measuring mRNA expression levels LightCycler 480 SYBR Green I Master (Roche Diagnostics, Germany) and LightCycler 480 System (Roche Molecular Systems Inc, Pleasanton, CA) were used. List of primer sequences for SYBR green analysis is included in Supplementary Table 1. Reaction volume was 20μL (2μL cDNA with 18μL of SYBR green master mix and corresponding primers 2 μM) and reactions were performed in triplicates. All transcription levels were normalized to β-actin levels.

### Statistical analysis

Statistical analysis was performed using Prism 9 (GraphPad Software, Inc., La Jolla, CA). One-way ANOVA followed by Tukey's multiple comparisons test was used as test of significancy for weight change in mice fed with high fat diet in treated and sham groups compared to baseline. Student’s T-test was used to test significancy of difference in weight loss and expression level of pro-inflammatory cytokines in adipose tissue samples in mice between slurry treated compared to sham group. *P* < 0.05 was considered significant. Data are presented as mean ± standard deviation.

## Results

### Slurry induces cryolipolysis in VAT and subsequent weight loss in mice

**Figure 1:**
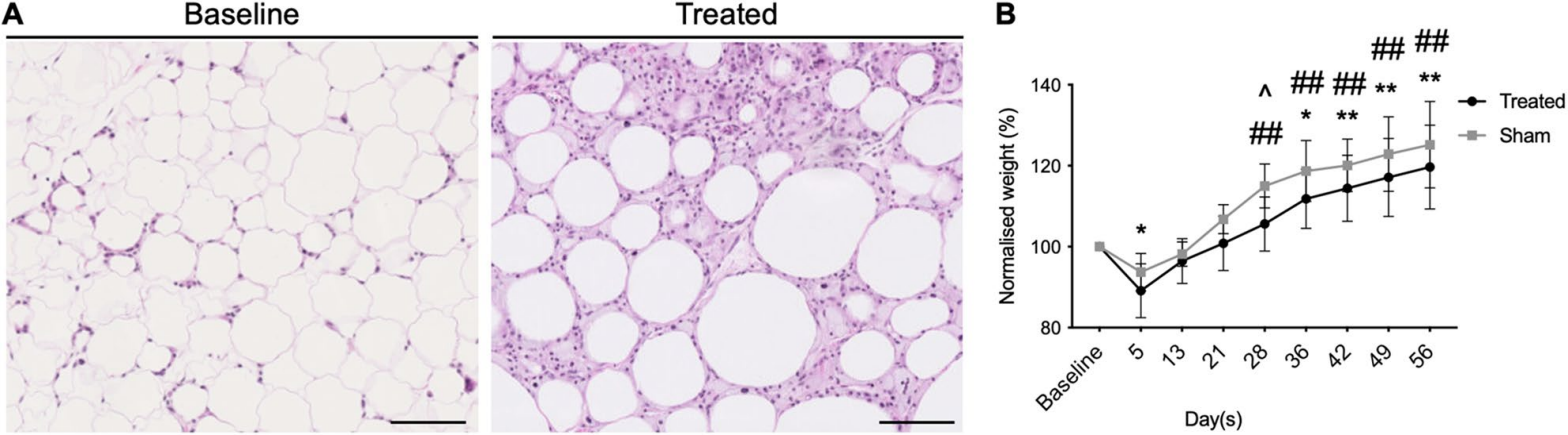
Slurry induces cryolipolysis of visceral adipose tissue and weight loss. (**A**) Representative images of adipose tissue in mice fed with high-fat diet at baseline and at day 21 post slurry treatment showing panniculitis around dying adipocytes; scale bar, 100 μm. (**B**) Graph shows weight change in mice fed with high fat diet in treated and sham groups. Data are presented as mean ± SD. n = 5 per group; * *P* < 0.05, ** *P* < 0.001 compared to baseline in slurry treated group and # *P* < 0.05, ## *P* < 0.001 compared to baseline in sham group by repeated measure one-way ANOVA followed by Tukey’s multiple comparisons test. ^ *P* < 0.05 slurry treated compared to sham group by Student’s T-test.

Slurry treatment was tolerated well in mice. Mice did not demonstrate any clinical signs of distress, skin damage, bleeding or infection. Histological analysis of adipose tissue taken at day 21 post treatment was consistent with cryolipolysis, notable for panniculitis as previously seen in subcutaneous adipose tissue (Fig. 1A). Mice were fed with high-fat diet during the study to avoid any confounding bias due to diet change. Mice fed with high-fat diet are expected to have continued increase in body weight over time. Body weight in mice treated with slurry decreased significantly at 5 days post treatment with gradual increase to baseline level (Fig. 1B). Control mice treated with the same procedure, but using room temperature (RT) control solution (sham group) did not show significant decrease in body weight post treatment. Body weight of mice in slurry treatment group were significantly less than the sham group only at day 28 post treatment when normalized to its baseline (105.61% ± 6.68% vs. 114.99% ± 5.42%; *p* < *0.05*) and were not seen at later time points. It took 36 days for the slurry treated animals to achieve weight gain that was significantly higher than baseline, in contrast to 28 days for the control RT solution treated mice to obtain weight gain significantly higher than the baseline (Fig. 1B).

### Injection of slurry induces cryolipolysis in VAT in rats

Histology analysis in samples taken at different time points post slurry injection in rats showed changes consistent with cryolipolysis, marked by panniculitis with abundance of inflammatory cells including lipid-laden macrophages, followed by normal wound healing process with evidence of new collagen deposition as previously described in subcutaneous fat treatments (Supplementary Fig. 1)^29^.

### Injection of slurry does not affect blood chemistry

Blood chemistry analysis showed no adverse effect on level of alkaline phosphatase (ALP), Alanine transferase (ALT), blood urea nitrogen (BUN), creatinine, total cholesterol, and triglyceride after injection of slurry at any of the time points tested when compared to baseline (Table 1). Since these rats were on high-fat diet they did have elevated baseline levels of ALP and triglyceride which did not change with treatment (Table 1).

**Table 1:** Blood chemistry in rats injected with slurry. n = 2–3 per group.

Timepoint	ALP (U/l)	ALT (U/l)	BUN (mg/dl)	Creatinine (mg/dl)	Total cholesterol (mg/dl)	Triglyceride (mg/dl)
Baseline	377.35 ± 123.49	34.00 ± 6.34	20.81 ± 7.38	0.49 ± 0.24	83.18 ± 14.19	202.59 ± 76.65
24 h	226.67 ± 92.59	107.00 ± 19.00	31.80 ± 20.03	0.67 ± 0.25	87.00 ± 18.49	137.00 ± 43.69
3 d	145.67 ± 50.68	30.33 ± 2.05	14.47 ± 3.72	0.53 ± 0.05	99.67 ± 14.97	86.00 ± 25.67
7 d	203.33 ± 81.79	35.67 ± 5.44	21.17 ± 3.47	1.00 ± 0.08	70.33 ± 13.10	189.67 ± 49.37
14 d	408.67 ± 31.94	29.5 ± 1.50	22.90 ± 1.94	0.43 ± 0.05	80.00 ± 12.33	221.33 ± 79.37
1 m	398.00 ± 92.00	39.33 ± 1.70	36.50 ± 13.00	1.10 ± 0.7	72.67 ± 4.78	173.00 ± 21.74
2 m	357.33 ± 52.50	37.67 ± 0.47	24.53 ± 3.03	0.43 ± 0.09	87.33 ± 9.84	188.67 ± 92.40
2 m UT	404.00 ± 51.00	44.00 ± 8.00	33.00 ± 3.30	0.70 ± 0.10	92.50 ± 6.50	162.50 ± 9.50

### RNA Sequencing profile of adipose tissue after injection of slurry

**Figure 2:**
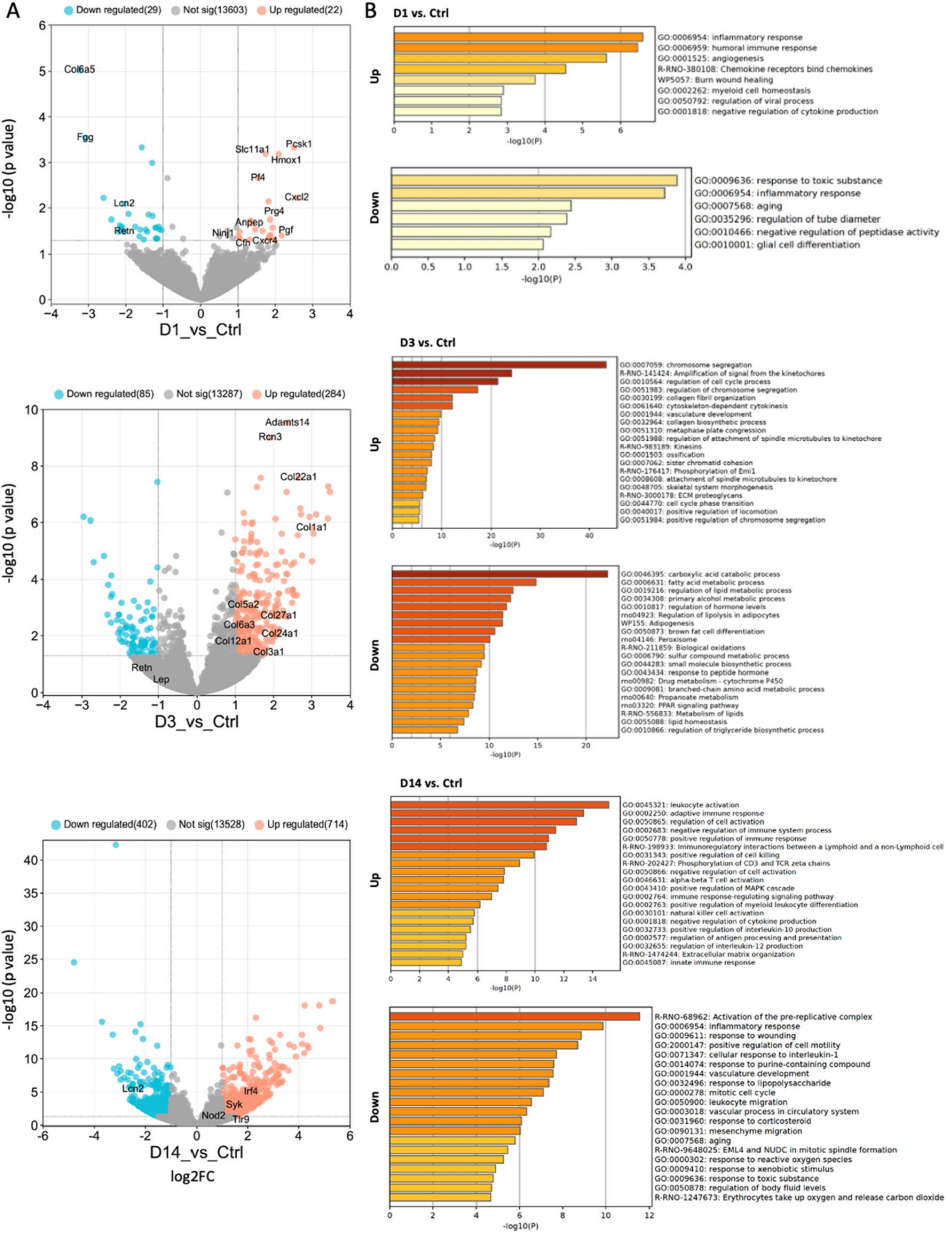
(**A**) RNA-seq was performed on control, D1, D7 and D14 rats. Volcano plots demonstrate differentially expressed genes (red data points) in each comparison. (**B**) Gene Ontology of differentially expressed genes.

RNA-sequencing was performed on samples collected at day 1, day 3 and day 14 post treatment to analyze changes in transcriptional profiles with slurry treatment. Principle Components Analysis (PCA) showed that RNA-seq libraries are clustered into different groups based on days post slurry injection (Supplementary Fig. 2). The number of differentially expressed genes (> 2-fold, FDR < 0.05) between untreated control adipose tissue versus slurry treated adipose tissue at day 1, day 3 and day 14 post treatment were 59, 1185, and 1116, repectively (Fig. 2A and Supplementary Table 2).

On the basis of gene ontology analysis, genes involved in inflammatory and immune response (GO:0006954) were the most upregulated genes at day 1 post treatment (Fig. 2B and Supplementary Fig. 3). Among these genes are Hmox1 (Heme Oxygenase 1), Cxcl2 (C-X-C Motif Chemokine Ligand 2), Slc11a1 (Solute Carrier Family 11 Member 1), Pf4 (Platelet factor 4), and Pcsk1 (Proprotein Convertase Subtilisin/Kexin Type 1) (Fig. 2A and Supplementary Fig. 3). Notably, Pcsk1 and Hmox1 play important roles in preventing obesity and metabolic disease^30,31^. Hmox1 is also involved in angiogenesis (GO:0001525) along with Ninj1 (Ninjurin 1), Cxcr4 (C-X-C chemokine receptor type 4), Anpep (Membrane alanyl aminopeptidase), Pgf (Placental Growth Factor), and Cfh (Complement factor H) and wound healing (GO:0042060) with Cxcl2, Cfh, Slc11a1, Pf4 (Fig. 2A and B). Other highly expressed genes at day 1 post treatment were genes involved in Chemokine and chemokine receptor interaction (R-RNO-380108) including Prg4 (Proteoglycan 4) (Fig. 2A and B). Highly downregulated genes at day 1 included genes associated with obesity including Col6a5 (collagen type VI alpha 5 chain), Fgg (Fibrinogen Gamma Chain), and adipokines Lcn2 (Lipocalin 2) and Retn (resistin) (Fig. 2A)^32–34^.

On day 3 post slurry treatment cellular responses such as fatty acid metabolic process (GO:0006631), regulation of lipolysis (rno04923) and triglyceride biosynthetic process (GO:0010866) in adipocytes were significantly downregulated (Fig. 2B). Among most downregulated genes were adipokines including Lep (leptin), Retn (resistin), and RBP4 (retinol binding protein 4) (Fig. 2A). Processes such as regulation of cell cycle (GO:0010564), regulation of cell division (GO:0051302), vasculature development (GO:0001944) and angiogenesis (GO:0001525) were upregulated at day 3 (Fig. 2B). In addition, there was significant upregulation of genes involved in collagen biosynthesis and organization (GO:0032964 and GO:0030199) including genes such as Col1a1 (collagen type I alpha 1 chain), Col3a1 (collagen type III alpha 1 chain), Col5a2 (collagen type V alpha 2 chain), Col6a3 (collagen type VI alpha 3 chain), Col12a1 (collagen type XII alpha 1 chain), Col22a1 (collagen type XXII alpha 1 chain), Col24a1 (collagen type XXIV alpha 1 chain), Col27a1 (collagen type XXVII alpha 1 chain) (Fig. 2A and B and Supplementary Fig. 3). Among most highly expressed genes at this time point was Adamts14 (a disintegrin and metalloproteinase with thrombospondin type 1 motif 14) which is involved in collagen fibril organization (GO:0030199) and Rcn3 (reticulocalbin 3) that is involved in collagen biosynthesis (GO:0032964) along with Col1a1 and Col22a1 (Fig. 2A and B).

On day 14 post treatment which is the peak of inflammatory response post slurry injection, there was significant upregulation in expression of genes involved in regulation of immune response (GO:0002683, GO:0050778, and GO:0002764), positive regulation of cell killing (GO:0031343), negative regulation of cytokine production (GO:0001818) (Fig. 2B). Notably, there was significant upregulation of genes involved in positive regulation of interleukin-10 production (GO:0032733) including Syk, Irf4, Nod2, Tlr9 (Fig. 2A and B). Among genes with downregulated expression at day 14 were genes involved in cellular response to interleukin-1 (GO:0071347) including IL-6 (Fig. 2A and B). In addition, there was downregulation of adipokine Lcn2 (Lipocalin 2) at day 14 (Fig. 2A).

### Slurry induces cell death and increased levels of pro-inflammatory cytokines in adipose tissue

**Figure 3:**
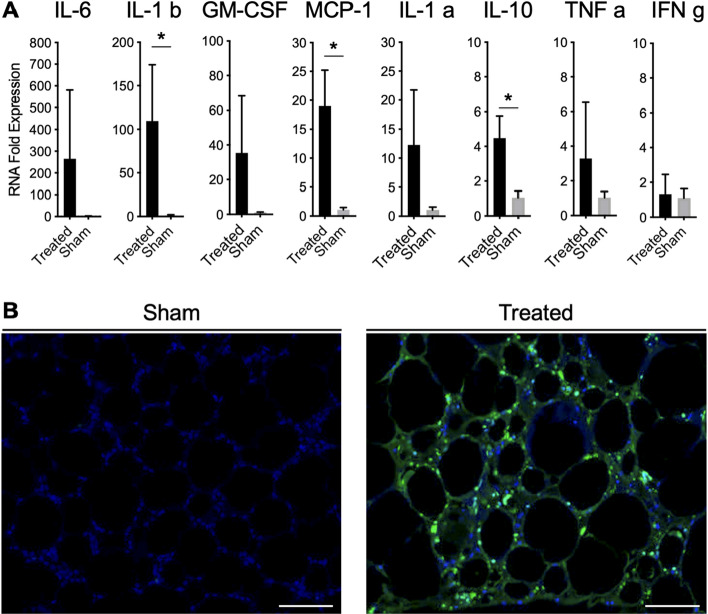
Slurry Induces cell death in adipocytes. (**A**) Graphs show expression level of pro-inflammatory cytokines in adipose tissue samples of slurry treated group at day 1 post treatment. Data are presented as mean ± SD. n = 3–4 per group; * *P* < 0.05 compared to baseline values by Student’s T-test. (**B**) Representative images of TUNEL stained adipose tissue samples in mice in slurry treated and sham groups at day 1 post treatment. TUNEL positive nuclei fragments are marked in green; scale bar, 100 μm.

Mice in slurry treatment group showed increased level of the pro-inflammatory cytokines that are known to play role in inflammatory response in adipose tissue and subsequent normal wound healing including IL-6, IL-1 b, GM-CSF, MCP-1, IL-1 a, IL-10, and TNF a in perigonadal adipose tissue at day 1 post treatment in comparison with the sham group, consistent with RNA-Seq data (Fig. 3A).

To examine if expression of these pro-inflammatory markers was associated with adipocyte cell death, TUNEL staining was performed. Slurry treatment induced adipocyte cell death, marked by presence of TUNEL positive nuclei at day one post treatment in mice (Fig. 3B), which was not seen in the RT control solution treated mice.

## Discussion

In this study ice slurry injection was a safe and effective method to produce cryolipoysis for selective reduction of VAT, leading to weight loss. This is the first report of successful use of ice slurry for targeting VAT and inducing weight loss. Furthermore, ice slurry treatment induced visceral adipocyte cell death as early as day 1 post treatment, associated with increased expression of pro-inflammatory markers in adipose tissue, followed by significant upregulation of genes involved in neocollagenesis and angiogenesis at later time points. Since VAT is strongly associated with obesity and metabolic syndrome, the results of this study offer the possibility of injecting slurry into abdominal VAT for targeted reduction thus improving not only weight but possibly also metabolic disease. Many more safety and feasibility trials will be required in large animals to demonstrate that injection of slurry into abdominal VAT will not pose a risk to surrounding organs or vessels. Adding image guidance such as ultrasound can help reduce the challenges associated with precise needle placement into abdominal VAT. Given that slurry is selective to targeting only adipose tissue makes this a potentially viable treatment.

Slurry-induced cryolipolysis in VAT is consistent with our previous findings of subcutaneous adipose tissue reduction with slurry treatment^24^. Histological analysis showed inflammatory infiltrate in VAT consistent with panniculitis (Fig. 1A). There was abundance of inflammatory cells including lipid-laden macrophages at day 7 followed by normal wound healing process that is similar to histological changes in subcutaneous adipose tissue (Supplementary Fig. 1)^24^.

Cryolipolysis of VAT by slurry induced weight loss in high-fat diet fed mice (Fig. 1B) which was not sustained, likely because mice were treated with slurry only once and continued on high-fat diet throughout the duration of the study. In a clinical setting, patients would be able to have multiple treatments and will likely be advised to modify their diet after treatment to allow for more sustained weight loss after VAT reduction. Future studies are required to determine the efficacy of multiple treatments.

We speculate the weight loss to be in part caused by reduced VAT mass itself which is supported by our finding of adipocyte cell death marked by positive TUNEL staining in slurry treated VAT. In addition, changes in expression of biologically active adipokines secreted by VAT could be another contributing factor in causing weight loss. Adipokines are peptides produced by adipose tissue that are known to systemically affect body weight by regulating the metabolism of tissues and organs^35^. Examples of adipokines are leptin, adiponectin, resistin, visfatin, and retinol-binding protein^36^. Obese patients have increased level of adipokines secreted by excess VAT volume and decreased expression of these adipokines by reduction of VAT is known to improve metabolic profile^35^. To investigate the mechanism of slurry induced reduction of VAT, RNA-sequencing was performed on rat perigonadal fat samples collected after slurry treatment and compared to untreated controls. We found decreased expression of multiple adipokines at different time points post slurry treatment. At day 1, resistin and lipocalin were downregulated; at day 3 there was decreased expression of resistin, leptin and retinol-binding protein and at day 14 lipocalin was downregulated.

In addition to adipokines, increased expression of genes such as Pcsk1 and Hmox1, which play important roles in preventing obesity and metabolic disease, could potentially contribute to reducing the risk of metabolic disease post slurry treatment^30,31^. Pcsk1 that is increased as early as day 1 after slurry treatment encodes a protease named prohormone convertase 1/3 that in turn activates prohormones including proinsulin and proglucagon. Level of Pcsk1 expression in adipose tissue is generally low and it is mostly expressed in neural and endocrine tissues^37^. Patients with genetic Pcsk1 deficiency experience obesity due to decreased proinsulin processing^30^, and a recently FDA-approved medication named setmelanotide is used as treatment for these patients^38^. Hmox1that is increased after injection of slurry is a heme oxygenase and antioxidant which maintains insulin sensitivity and its deficiency causes metabolic disease^31^. Moreover, some of the down-regulated genes including Col6a5 and Fgg are associated with obesity. Col6a5 codes collagen type VI alpha 5 chain. Expression of Col6a5 in adipose tissue is associated with obesity, and insulin resistance^32^. Fgg that codes fibrinogen gamma chain was another gene with decreased expression that is associated with diet induced obesity^33^.

In a recent study in which authors applied topical cooling on VAT in a swine model of metabolic disease, it was reported that the cooling of VAT induced 30% loss in mesenteric fat volume, improved insulin sensitivity and blood pressure in this model^39^. Although authors of this study did not show weight loss or check for changes in expression of adipokines and genes associated with obesity that led to improved metabolic disease profile in these swine, this study provides proof for the notion that reduction of VAT with cooling in this swine model does promote concomitant reduction in insulin resistance and metabolic syndrome risk. Our findings provide a potential new and practical method of selective and targeted reduction of viscleral fat that can lead to weight loss and improved methabolic disease profile. Unlike topical cooling, injectable slurry can make such procedures minimially invasive and less time consuming to perform.

Studying the mechanism of slurry induced cryolipolysis showed increased expression of genes regulating the inflammatory and fibrotic responses at different stages of wound healing process. These findings are consistent with slurry induced adipocyte cell death that is associated with increased level of pro-inflammatory cytokines including IL-6, IL-1 b, GM-CSF, MCP-1, IL-1 a, IL-10, and TNF a in q-PCR data at day 1. These pro-inflammatory cytokines are needed for influx and differentiation of inflammatory cells including macrophages in adipose tissue that promote clearance of cell debris, and initiate normal wound healing^40^. Slurry induced adipocyte cell death explains the presence of lipid-laden macrophages in VAT after slurry treatment seen on histology which is consistent with our previous findings of slurry injection in subcutaneous adipose tissue^24^. Consistent with the normal wound healing process, slurry treatment turned on neocollagenesis as early as day 3 post treatment as seen in the RNA-Seq results. We had previously reported that neocollagenisis was present after ice-slurry treatment of subcutaneous adipose tissue, supporting the reduced laxity of tissue seen after cryolipolysis^29^. Increased expression of Col1a1 has been reported as a result of cryolipolysis of subcutaneous adipose tissue using topical cooling^41^. Among the genes upregulated by slurry treatment, presence of Prg4 was noticeable as early as day 1. Prg4 is a proteoglycan that enhances connective tissue regeneration while suppressing scar formation^42^. This finding hints on controlled neocollagenesis caused by slurry without risk of scar formation. Similar to the controlled wound healing process, immune response following slurry treatment shows a controlled pattern. The inflammatory response peaked at day 14 as seen on histology (Supplementary Fig. 1), which corresponded with significant changes in expression of genes involved in regulating immune response in RNA-seq data. Most importantly, there was upregulation of genes involved in positive regulation of IL-10, including Syk, Irf4, Nod2, Tlr9 at day 14 post slurry treatment to maximize the level of IL-10 that had already started to rise as early as day 1^43^. IL-10 is the cytokine with strong anti-inflammatory properties that plays a crucial role in supressing host immune response and preventing damage to the host and maintaining normal tissue homeostasis^43^. The peak in expression of IL-10 at day 14 post-slurry treatment likely leads to the controlled supression of the immune response which is critical for avoiding a chronic inflammatory state.

Treatment of VAT with slurry, similar to subcutaneous adipose tissue^24^, did not induce any observable local or systemic side effects or lab chemistry abnormalities. The safety of the slurry was confirmed by a recent human study in which slurry was safely injected into subcutaneous adipose tissue^25^. Although more studies are needed to investigate the safety of using slurry to target fat in other areas of the body such as visceral fat, the data from this study and the pre-existing human safety study for subcutaneous adipose tissue, make the translation of our technology to human patients for reducing VAT possibly attainable in near future. We speculate that contraindications for using large volumes of injectable slurry could include chronic kidney disease and congestive heart failure as slurry consists of a hypertonic solution. Other contraindications are known history of cold sensitivity, cryoglyobulenima, and cold induced vasculitis. A limitation of our study was that we used a surgical approach to expose VAT for slurry treatments since we were working with small animal models that make injections of 10ml slurry challenging. However, in patients, we envision the use of laparoscopy or image guidance to inject or infuse ice slurry directly into the intra-abdominal cavity to selectively target and reduce intra-abdominal VAT. We have previously shown that due to the presence of ice particles, slurry can easily be visualized using ultrasound^29,44^. Although ice slurry is a selective treatment, the safe placement of the needle into the abdominal VAT while avoiding any injury by the needle to important anatomic structures will require further testing and development. In our future study, we plan to examine the feasibility of slurry injection in visceral fat under ultrasound guidance in large animal models.

## Conclusion

In this study we showed that the initial safety, feasibility and mechanism of ice slurry-induced cryolipolysis of VAT in rodents. Although more studies will be needed in large animals to demonstrate the safety and feasibility of this approach, slurry could potentially serve as a novel method for safe and selective reduction of abdominal VAT in obese patients that cannot tolerate current obesity treatment methods.

### Supplementary Information

Supplementary Information 1.

Supplementary Information 2.

Supplementary Information 3.

Supplementary Information 4.

Supplementary Information 5.

## Data Availability

All data are available in the main text or supplementary materials. RNA-Seq data have been deposited in the Gene Expression Omnibus (GEO) with the accession number of GSE240955.
