# Supplementary material for: Selective reduction of visceral adipose tissue with injectable ice slurry
Source: Sci Rep. 2023 Sep 28;13:16350. doi: 10.1038/s41598-023-43220-9 (PMC10539385; doi:10.1038/s41598-023-43220-9)
Supplement: Supplementary file 1 — Supplementary Information 1. [file 41598_2023_43220_MOESM1_ESM.docx]

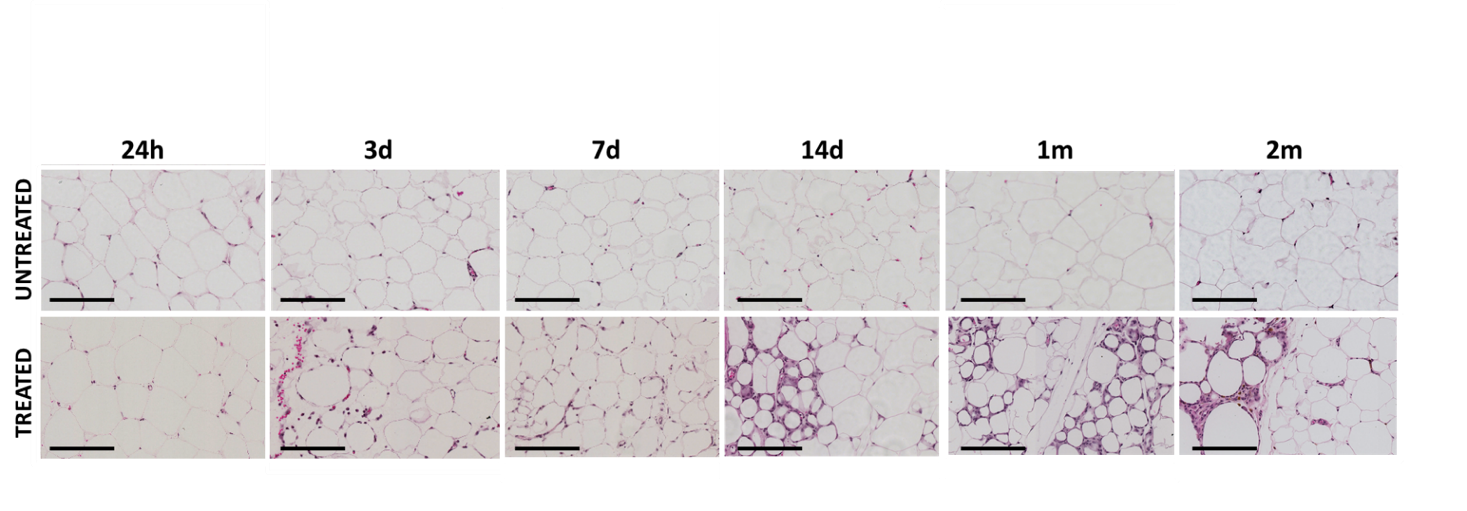


Supplementary Figure 1. Injection of slurry induces cryolipolysis of visceral adipose tissue. Representative images of adipose tissue in rats fed with high-fat diet at baseline and post slurry treatment; scale bar, 100 μm.
