# Supplementary material for: Selective reduction of visceral adipose tissue with injectable ice slurry
Source: Sci Rep. 2023 Sep 28;13:16350. doi: 10.1038/s41598-023-43220-9 (PMC10539385; doi:10.1038/s41598-023-43220-9)
Supplement: Supplementary file 2 — Supplementary Information 2. [file 41598_2023_43220_MOESM2_ESM.docx]

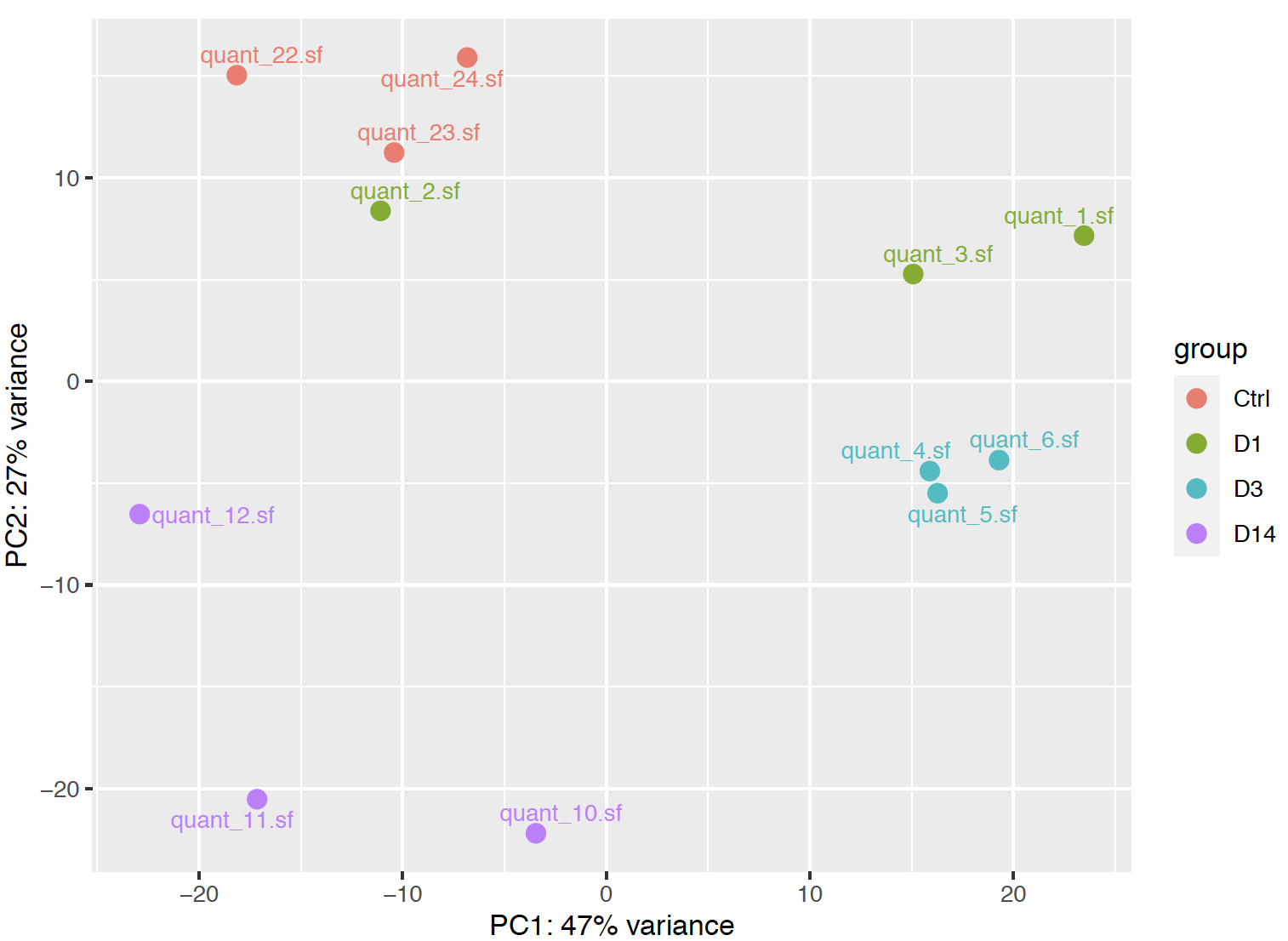


Supplementary Figure 2. Principle Components Analysis (PCA) showed that RNA-seq libraries are clustered into different groups based on days post slurry injection. Each dot represents one library. Replicates from the same group are highlighted with the same color.
