# Supplementary material for: Selective reduction of visceral adipose tissue with injectable ice slurry
Source: Sci Rep. 2023 Sep 28;13:16350. doi: 10.1038/s41598-023-43220-9 (PMC10539385; doi:10.1038/s41598-023-43220-9)
Supplement: Supplementary file 3 — Supplementary Information 3. [file 41598_2023_43220_MOESM3_ESM.docx]

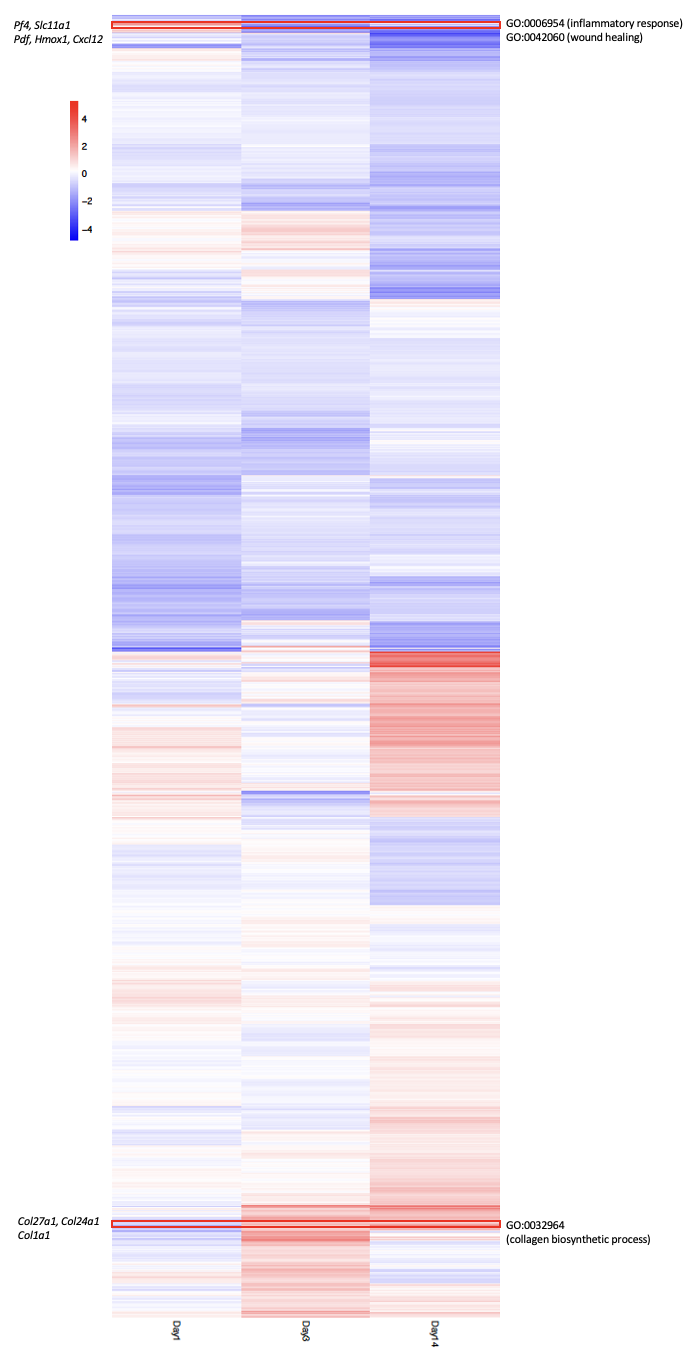


Supplementary Figure 3. Heatmap of all annotated genes. Genes involved in inflammatory response are upregulated at D1 and downregulated at D14 while genes involved in collagen biosynthesis process are upregulated at D3.
