# Supplementary material for: Selective reduction of visceral adipose tissue with injectable ice slurry
Source: Sci Rep. 2023 Sep 28;13:16350. doi: 10.1038/s41598-023-43220-9 (PMC10539385; doi:10.1038/s41598-023-43220-9)
Supplement: Supplementary file 4 — Supplementary Information 4. [file 41598_2023_43220_MOESM4_ESM.docx]

Supplementary Table 1. RT-PCR Primer sequences

| Gene Primer | Sequences (5′ → 3′) |
| --- | --- |
| *IL-6* | F: GCTACCAAACTGGATATAATCAGGA  R: CCAGGTAGCTATGGTACTCCAGAA |
| *IL-1b* | F: TCTAACTGGCAAACCCAAACTT  R: AGTCCCACTGTCCGTCTCAAT |
| *GM-CSF* | F: GGCCTTGGAAGCATGTAGAGG  R: GGAGAACTCGTTAGAGACGACTT |
| *MCP-1* | F: GTGTTGGCTCAGCCAGATGC  R: GACACCTGCTGCTGGTGATCC |
| *IL-1a* | F: GCACCTTACACCTACCAGAGT  R: AAACTTCTGCCTGACGAGCTT |
| *IL-10* | F: GCTCTTACTGACTGGCATGAG  R: CGCAGCTCTAGGAGCATGTG |
| *TNF α* | F: ACCACGCTCTTCTGTCTACTGA  R: TCCACTTGGTGGTTTGCTACG |
| *IFN g* | F: ATGAACGCTACACACTGCATC  R: CCATCCTTTTGCCAGTTCCTC |
| *β-actin* | F: CCTTCTTGGGTATGGAATCCTGT  R: CACTGTGTTGGCATAGAGGTCTTTAC |
